# Is Radiosensitivity Associated to Different Types of Blood Groups? (A cytogenetic study)

**Published:** 2013

**Authors:** Farideh Elahimanesh, Ali Shabestani Monfared, Meysam Khosravifarsani, Haleh Akhavan Niaki, Zeinab Abedian, Karimollah Hajian-Tilaki, Sajad Borzouisileh, Nayer Seyfizadeh, Mehrangiz Amiri

**Affiliations:** 1 *Cellular & Molecular Biology Research Center (CMBRC), Babol University of Medical Sciences, Babol, Iran.*; 2 *Department of Radiology, Faculty of Medicine, Kurdestan University of Medical Sciences, Kurdestan, Iran.*; 3 *Department of Clinical Biochemistry, Faculty of Medicine, Tabriz University of Medical Sciences, Tabriz, Iran.*

**Keywords:** Radiosensitivity, blood group, CBMN assay

## Abstract

Many biological factors affect radiosensitivity. In this study, radiosensitivity among the different blood groups was investigated. Peripheral blood sample of 95 healthy people were divided into two parts. One part was irradiated with 2 Gy Co-60 gamma rays and the second one was considered as control. Then all the samples were studied by cytokinesis-blocked micronucleus assay (CBMN assay). Our study showed that the radiosensitivity index of A+ and O+ groups was significantly higher and lower than other blood groups, respectively. It seems that blood type can be used as a radiosensitivity index for determining the given dose to radiotherapy, although extensive studies are necessary.

Radiosensitivity is the relative susceptibleness of cells, tissues, organs or organisms to the dangerous effect of ionizing radiation (1). Inherent characteristic is one of the important reasons of differences in radiation sensitivity (1-2). The physical specifications of ionizing radiation such as its type (particle or photon), energy and dose rate could alter the biological response of organ or tissue to ionizing radiation (2-4). Previous studies have confirmed the relationship between genetics and radiosensitivity (5-6). A recent study has shown that mutations in the ataxia telangiectasia gene (ATM) result in an abnormal p53-mediated cellular response to DNA damage produced by ionizing radiation. Also, the potential role of several identified genes such as BRCA and NBS1, which are involved in the cellular response to radiation induced DNA damage is reported too (6). A clinical study has suggested that a large spectrum of normal tissue reactions may occur among the radiotherapy patients. Because of the difference in the individual radiosensitivity and radiotherapy, the patients who receive identical dose have different normal tissue reactions varying from undetectable to severe (7). People with higher radiosensitivity, most likely will suffer from deterministic and stochastic effects in radiotherapy (7). The results of the studies have revealed that over expression of KU80 gene is an important factor for predicting radiosensitivity in the head and neck cancers (5). It has shown an association between the *in vitro* radiosensitivity of breast cancer patients and the clinical incidence of late (e.g. fibrosis, telangiectasia) normal tissue reaction to radiotherapy (6). In addition to biological conditions, environmental conditions such as existence of radiosensitizers and radioprotectors undoubtedly affect the biological damage of ionizing radiation. One well-studied example is the presence of oxygen during exposure to ionizing radiation that stabilizes reactions to ionizing radiation and then increases the biological damage of radiation (4). The blood group is an inherent characteristic and its classification is based on the presence or absence of ABO blood group antigens on the surface of red blood cells. The occurrence of some diseases is related to blood type (8) and studies have reported that ABO blood group is an important genetic risk factor for several radiation related illnesses such as pancreatic cancer (9), hepatocellular carcinoma (10), endometrial and cervical cancer (11). In this study, the association between the radiosensitivity and ABO blood group was investigated by cytokinesis-blocked micronucleus assay (CBMN) in a case-control cytogenetic study.

## Materials and Methods

**Subjects and sampling**

Ten milliliter blood samples of 95 (25 A+, 25 B+, 25 O+ and 20 AB+) non-radiation worker, non-smoker or alcohol-user healthy donors age between 18-25 years were taken under sterile conditions in the presence of sodium heparin anticoagulant. The samples were divided into two identical values (5ml) which were maintained in similar conditions. The subjects’ blood groups, any cancer history in their families and recent radiation exposures were filled in the questionnaire through an interview.

**Irradiation**

One part of each sample was considered as the control and the second equivalent part was exposed to 2 Gy of gamma rays from a tele-cobalt therapy source (Theratone780, Canada). The dose rate was 120 cGy/min and the source to samples distance (SSD) was 80 cm. The exposed and non-exposed blood samples were transferred to cell culture laboratory for the CBMN assay.

**CBMN (cytokinesis blocked micronuclei assay)**

CBMN assay was performed on both exposed and control samples as reported by international atomic energy agency (IAEA). In this cytogenetic technique, 0.5 ml of the whole blood was added to 4.5 ml culture medium (RPMI 1640) supplemented with fetal calf serum, 1% L-glutamine and antibiotics. Then 100 µl phytohaemagglutinin (SIGMA) diluted in PBS was added as mitogen. The sample was incubated at 37º for 44 h then 100 µl cytochalasin B (6 µg/ml diluted in DMSO) was added for cessation of the cytokinesis in the binucleus state. The binucleated lymphocytes were harvested 28 h later. The samples were centrifuged at 2000 rpm for 10 min (BOECHO U-320 R) and the supernatant was discarded. The pellet remained at the bottom of tubes was treated with 2-3 ml of fresh hypotonic solution (0.075 M KCl) and then centrifuged at 1200 rpm for 7 min. After discarding the supernatant, 5 ml of the fixing solution (methanol:glacial acetic acid 6/1) was added quickly. After 20 min, the tubes were centrifuged (1200 rpm for 7 min) and the fixation was repeated three times at 1200 rpm for 7 min. Subsequently, the cells were dropped on clean slides and stained with Giemsa solution (Giemsa stock: PBS, 1/10) for 10 minutes. The slides were washed with distilled water and were dried by air. All the slides were studied under a light microscope in 40× magnification using SAIRAN microscope. The slides were coded before analyzing for blinding purpose. The Micronuclei were scored in 1000 binucleated (BN) cells and scoring was blinded according to the scoring scale suggested by Fenech (12-13). The proportion of MN in exposed to non-exposed samples in each blood group was considered as its radiosensitivity index (1, 3). If one person's cells are more radiosensitive, after taking 2 Gy radiation dose, more DNA breaks (or MNs) occurs and its proportion to non exposed cells will be higher than a person with lower radiosensitivity. 

Statistical analysis

The statistical analysis was performed using SPSS 16 by Pair sample t-test between the control and exposed groups and analysis of variance (ANOVA) test between the different blood groups. The p-value < 0.05 was considered statistically significant.

## Results

**Fig. 1:**
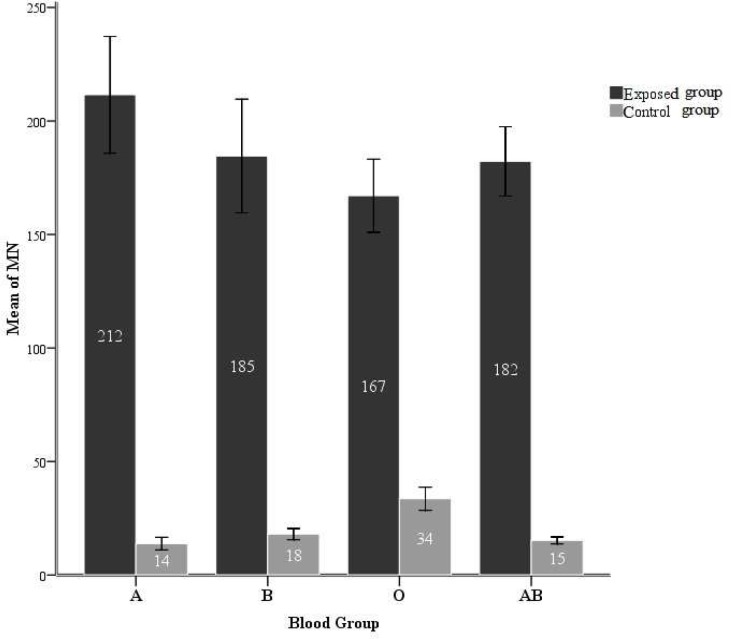
Mean frequency of micronuclei in control and exposed groups of different blood groups

The mean micronuclei frequencies of the different blood groups were shown in figure 1. As the graphs show, the micronuclei frequency in the exposed samples for all of the blood groups is significantly higher than the control (P <0.001). Also, there is a significant difference in the micronuclei frequencies of O+ and other blood types in control group (p< 0.001). The difference of micronuclei frequencies between A+ and O+ in exposed groups is significant too (P= 0.015).

The increase in the number of micronuclei after exposure to 2 Gy irradiation for all of the four blood groups are shown in table 1. As quoted in this table, the highest and lowest increase was seen in A and O blood groups, respectively. Also this parameter was significantly different between A and O blood groups.

## Discussion

CBMN assay is the standard technique for measuring the human population micronuclei and the estimation of absorbed dose for the prediction of deterministic and stochastic effects in nuclear accident (7). It is a cytogenetic method for the evaluation of cytotoxic effects of chemical materials and ionizing radiation in mammalian system too. *In vitro* cytokinesis blocked micronuc-leus assay can be used for the prediction of radiosensitivity of the tumor cells and as an index for the inter-individual differences in radio-sensitivity (13).

Our data clearly indicate that the mean frequency of MN in exposed groups is remarkably higher than the control groups. These findings are in agreement with the previous cytogenetic inves-tigations which have been done by Maffie et al. (14), Thierens (15) and Khosravifarsani et al. (1).

The recent study performed by Khosravi-farsani et al. in 2012 showed that radiosensitivity in left-handed is greater than right-handed breast cancer women. They also explained that radiosensitivity in left-handers is higher compared to right-handers (1). Our data suggest that A+ is the most radiosensitive and O+ have the lowest radiosensitivity among the studied blood groups. The results obtained from Garriga and Ghossein revealed that O blood group has greater radiation response than either blood type in carcinoma of cervix patients (16).

**Fig. 2:**
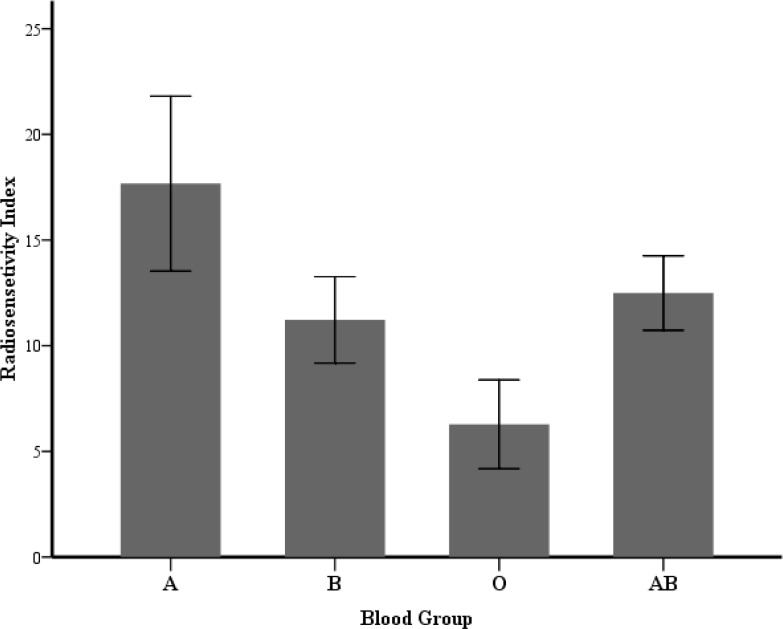
Radiosensitivity index (RI) of different blood groups

**Table 1:** Micronuclei number increase in blood samples exposed to 2 Gy irradiation

Mean	SD	Max	Min	N	Blood group
197.72	60.79	314	94	25	A
166.52	58.37	283	72	25	B
133.48	36.45	214	76	25	O
166.95	32.64	231	102	20	AB

A previous study performed by Dabelsteen et al. reported that alteration of the cell-surface is a factor in the development of malignancies (17). Earlier observations have declared that there is an association between various cancers and ABO blood groups (18-20). Stamatakos et al. showed that the frequency of ductal breast cancer is higher in A blood group (18). Tursen et al. have investigated the relationship between blood groups and skin cancer (19). They reported that although patients with A and O blood groups have the most and the lowest occurrence of skin cancer but this difference is not statistically significant. Wolpin et al. concluded that the frequency of pancreatic cancer in patients with O blood group was obviously lower than other blood groups (20). Doll et al. showed that the number of gastric ulcer and neoplasm in patients with A blood group were significantly higher than the other blood groups (21). You et al. indicated that the frequency of dysplasia and metaplasia and gastric atrophy in A blood group was higher than the blood groups (22). To date, no report has evaluated the association of blood groups and radiosensitivity.

The present study addresses more investiga-tions on the association of ABO blood groups and radiosensitivity. From a genetic point of view, the repair of double strand breaks generated by radiation involves two main mechanisms, non-homologous end-joining (NHEJ) and homologous recombination (HR). Our data suggest that there may be an association between some alleles of ABO blood groups and specific alleles of genes involved in DNA double strand breaks repair. Further molecular studies are needed to investigate candidate loci involved in the DNA repair systems on chromosome 9 near ABO locus at 9q34. If future research could prove our findings, blood groups could be used as a radiosensitivity index for determining the given dose to radiotherapy patients and protecting workers against exposure to ionizing radiation.
